# Mining of haplotype-based expressed sequence tag single nucleotide polymorphisms in citrus

**DOI:** 10.1186/1471-2164-14-746

**Published:** 2013-11-01

**Authors:** Chunxian Chen, Fred G Gmitter Jr

**Affiliations:** 1 University of Florida, IFAS, Citrus Research and Education Center, 700 Experiment Station Road, Lake Alfred, FL 33850, USA; 2 USDA, ARS, Southeastern Fruit and Tree Nut Research Laboratory, 21 Dunbar Road, Byron, GA 31008, USA

**Keywords:** Haplotype, Heterozygosity, Polymorphism, Transition, Transversion, Insertion/deletion, Non-synonymous, Synonymous

## Abstract

**Background:**

Single nucleotide polymorphisms (SNPs), the most abundant variations in a genome, have been widely used in various studies. Detection and characterization of citrus haplotype-based expressed sequence tag (EST) SNPs will greatly facilitate further utilization of these gene-based resources.

**Results:**

In this paper, haplotype-based SNPs were mined out of publicly available citrus expressed sequence tags (ESTs) from different citrus cultivars (genotypes) individually and collectively for comparison. There were a total of 567,297 ESTs belonging to 27 cultivars in varying numbers and consequentially yielding different numbers of haplotype-based quality SNPs. Sweet orange (SO) had the most (213,830) ESTs, generating 11,182 quality SNPs in 3,327 out of 4,228 usable contigs. Summed from all the individually mining results, a total of 25,417 quality SNPs were discovered – 15,010 (59.1%) were transitions (AG and CT), 9,114 (35.9%) were transversions (AC, GT, CG, and AT), and 1,293 (5.0%) were insertion/deletions (indels). A vast majority of SNP-containing contigs consisted of only 2 haplotypes, as expected, but the percentages of 2 haplotype contigs varied widely in these citrus cultivars. BLAST of the 25,417 25-mer SNP oligos to the Clementine reference genome scaffolds revealed 2,947 SNPs had “no hits found”, 19,943 had 1 unique hit / alignment, 1,571 had one hit and 2+ alignments per hit, and 956 had 2+ hits and 1+ alignment per hit. Of the total 24,293 scaffold hits, 23,955 (98.6%) were on the main scaffolds 1 to 9, and only 338 were on 87 minor scaffolds. Most alignments had 100% (25/25) or 96% (24/25) nucleotide identities, accounting for 93% of all the alignments. Considering almost all the nucleotide discrepancies in the 24/25 alignments were at the SNP sites, it served well as *in silico* validation of these SNPs, in addition to and consistent with the rate (81%) validated by sequencing and SNaPshot assay.

**Conclusions:**

High-quality EST-SNPs from different citrus genotypes were detected, and compared to estimate the heterozygosity of each genome. All the SNP oligo sequences were aligned with the Clementine citrus genome to determine their distribution and uniqueness and for *in silico* validation, in addition to SNaPshot and sequencing validation of selected SNPs.

## Background

Single nucleotide polymorphism (SNP) refers to an allelic single-base variation between two haplotype sequences in an individual or between any paired homologous chromosomes across homogenous members. SNPs are most abundant among genomic DNA variations and ubiquitous in both functional genes and non-coding regions [1]. Because they are conserved during evolution, associated with genetic traits, and suited for high throughput genotyping, SNPs are a popular and powerful tool for various genetics and genomics studies, such as mapping of whole genomes, tagging of important traits, comparison of genome evolution, classification of diverse clades, and many rapidly developing areas such as pharmacogenomics and functional proteomics [2-4]. These SNPs from expressed sequence tags (ESTs) represent hundreds of thousands of functional genes and likely control many genetic traits [5-8]. Due to degeneracy of most three-nucleotide genetic codons, a SNP in the coding regions may be synonymous (sSNP) if it does not result in change of the protein sequence or non-synonymous (nsSNP) if it does. The nsSNPs are usually more biologically relevant because the resulting amino acid changes in proteins may change their secondary structures and functions and cause phenotypic mutations [1,8,9].

SNP discovery usually is accomplished through computational alignment of redundant DNA sequences with each other or with a high-quality reference genome where discrepant nucleotides can be detected and evaluated. For the redundancy-based computational approach, in addition to sequencing errors as a source of false SNPs [5,7,10], it may be even more challenging to distinguish real SNPs among allelic sequences from single nucleotide discrepancies among highly identical paralogous sequences [8,11]. Several bioinformatics programs (pipelines) have been developed for automatic SNP mining, using different input data, computational algorithms, quality evaluation strategies, and/or output formats. For example, the PolyPhred and PolyBayes pipeline typically requires sequence trace files or extracted sequences with base calling quality values to minimize false SNPs resulting from sequencing errors [12-14]. PolyBayes also includes an extra implementation to identify paralogs and their derived false SNPs [13]. Others like autoSNP and QuailitySNP can accept sequences without quality files for initial redundancy-based detection, and then grade SNPs by confidence levels, which are more commonly used with public ESTs that usually do not have trace or quality files [8,15]. The QualitySNP pipeline implements a haplotype reconstruction algorithm and confidence scoring approach to detect reliable synonymous and non-synonymous SNPs from public ESTs without quality files and a reference genome [8]. In other words, it re-clusters ESTs in a contig to determine the potential haplotypes in the contig. Only single discrepant nucleotides between any two reconstructed haplotypes would be scored a potential SNP. Sequencing differences can also result from sequencing errors or alignment of paralogs. Only those potential SNPs passing additional confidence interrogation are identified as quality SNPs. Reliable quality SNPs represent the different alleles (haplotypes) of a gene. As opposed to low-confidence and false SNPs, the use of quality SNPs can benefit allele-trait association studies [8].

Most citrus species are diploid (2n = 2× = 18), with highly heterozygous and relatively small genomes and over 30,000 predicted genes [16]. In general, citrus refers to true biological species and ancestrally domesticated introgressions in *Citrus* and those in the sexually compatible *Fortunella* (kumquat) and *Poncirus* (trifoliate orange) genera. Citrus fruit types are diverse, and include sweet orange (*Citrus sinensis*), mandarin (*C. reticulata*), grapefruit (*C. paradisi*), lemon (*C. limon*), lime (*C. aurantifolia*), pummelo (*C. maxima*), and citron (*C. medica*). Each type consists of many cultivars primarily selected from spontaneous bud sports, chance seedlings, induced mutants, or conventional hybrids. It is widely believed that only *C. maxima*, *C. reticulata*, and *C. medica* are true species, although the binomial names for the other ancestral hybrid and introgression cultivars are widely accepted and used [17,18]. These citrus types likely vary in levels of heterozygosity and share alleles resulting from early introgressions across these genomes, according to SSR markers [19-21]. A haploid Clementine genome sequence was produced using Sanger technology, and one diploid sweet orange genome using Roche 454 technology [22], along many other citrus genomes using other re-sequencing platforms (Gmitter et al. unpublished data). Together with other available citrus genomic resources, it is now possible for SNP detection and comparison of large-volume citrus Sanger EST datasets within and among different citrus cultivars. These gene-based SNPs, once available for the citrus community, will be very valuable in many genetic and genomic studies, and helpful for trait-targeted breeding as well [20,21,23].

In this paper, SNPs in public ESTs from 27 different citrus genotypes were detected by the QualitySNP pipeline and compared to estimate the heterozygosity of each genome. All of the short SNP oligo sequences were also aligned with the Clementine citrus genome to determine their distribution and uniqueness in the genome and for in *silico* validation. Selected SNPs were also validated by SNaPshot and sequencing.

## Methods

### Citrus ESTs and cultivars

All citrus ESTs were retrieved from the National Center of Biotechnology Information (NCBI) EST database or ftp repository if available. There were 27 citrus cultivars or biotypes with ESTs (Table 1, Additional file 1). In addition to the binomial and common names, the abbreviations for 27 cultivars were designated to facilitate presentation (Table 1, Additional file 1); the binomial names are those used for the accessions in the NCBI database. ESTs were searched for SNPs using the QualitySNP pipeline [8] in each of the 27 cultivars and in three cultivar groups, 12 mandarins (M12), 7 limes/lemons/citron (L7), and all 27 cultivars (C27). The mining results for individual cultivars in the three groups were summed, giving SM12, SL7, and SC27, respectively used to compare with of M12, L7, and C27 (Additional file 1). 'Ridge Pineapple’ sweet orange (*Citrus sinensis*) was selected for SNP validation because the most ESTs and SNPs are from sweet orange and it is a parent to several widely used mapping populations.

**Table 1:** Public ESTs in citrus cultivars/biotypes

**No**	**Binomial names**	**Common names**	**Abbreviations**	**EST numbers**
**1**	***Citrus sinensis***	**Sweet orange**	**SO**	**213,830**
**2**	***C. clementina***	**Clementine mandarin**	**CM**	**122,005**
**3**	***C. reticulata***	**Ponkan mandarin**	**PM**	**52,340**
**4**	***C. unshiu***	**Satsuma mandarin**	**SM**	**19,072**
5	*C. reshni*	Cleopatra mandarin	LM	5,768
6	*C. sunki*	Hayata mandarin	HM	5,216
7	*C. tamurana*	Rixiangxia mandarin	RM	358
8	*C. hassaku*	Hassaku mandarin	KM	154
9	*C. natsudaidai*	Summer orange	UM	202
10	*C. reticulata x C. temple*	Orah tangor	OT	5,823
11	*C. clementina x C. reticulata*	Fortune tangor	FT	1,917
12	*C. nobilis x C. kinokuni*	Kankitsu Chukanbohon Nou 6 Gou tangor	KT	645
13	*C. sinensis x C. reticulata*	Amakusa tangor	AT	160
**14**	***C. limonia***	**Rangpur lime, Mandarin lime**	**ML**	**11,045**
**15**	***C. latifolia***	**Tahiti lime**	**TL**	**8,756**
**16**	***C. aurantifolia***	**Mexican lime**	**KL**	**8,219**
**17**	***C. limettioides***	**Palestine Sweet lime**	**SL**	**8,188**
18	*C. limon*	Lisbon lemon	LL	1,505
19	*C. jambhiri*	Rough lemon	RL	1,017
20	*C. medica*	Etrog citron	EC	1,115
**21**	***C. aurantium***	**Sour orange, Bitter orange**	**BO**	**14,584**
**22**	***C. paradisi***	**Grapefruit**	**GF**	**8,039**
23	*C. macrophylla*	Alemow pepada	AP	1,929
24	*C. paradisi x P. trifoliata*	Swingle citrumelo	SC	7,954
25	*C. sinensis x P. trifoliata*	Carrizo citrange	CC	1,837
26	*Fortunella margarita*	Nagami kumquat	NK	2,924
**27**	***Poncirus trifoliata***	**Trifoliate orange**	**TO**	**62,695**
	**2-13 combined**		**M12**	**213,660**
	**14-20 combined**		**L7**	**39,845**
	**1-27 combined**		**C27**	**567,297**

All ESTs are retrieved from the NCBI repository. Those in bold font are over 8,000 ESTs. All mandarin (No. 2–13) and lime/lemon (No. 14–20) types were listed together. The abbreviation for each cultivar and total was designated to facilitate presentation.

### SNP discovery and primer design

The QualitySNP pipeline was installed and used for SNP discovery, following the program manual and recommended parameters [8]. QualitySNP first identified haplotypes in a contig by re-clustering its ESTs and extracted all nucleotide discrepancies (called potential SNPs, pSNPs) between identified haplotypes in a contig, from which a subset of so-called quality SNPs (qSNPs) was identified based on allele and SNP confidence scores defined in the haplotype-based mining algorithm [8]. These qSNP-containing contigs and 25-mer oligo sequences, along with much other mining information, were saved in separate files for database construction and result summary. The ratios of qSNP/pSNP were calculated to indicate the percentage of nucleotide discrepancies (pSNPs) identified as high-qaality SNPs (qSNPs) by the QualitySNP algorithm. Bioinformatics programs included in the pipeline were cross_match in the phred-phrap-consed package [24,25] to remove vectors, CAP3 [26] to assemble ESTs, FASTY [27] to align ESTs to the proteins in the Uniprot database for identification of non-synonymous and synonymous SNPs. BatchPrimer3 [28] was used to design a forward (F), a reverse (R), and a single base extension (SBE) primer flanking each SNP site. The F, R and SBE primers of 96 SNPs from SO were selected for both sequencing and SBE genotyping validation (Additional file 2). After sorting by the lengths of SBE primers, except the first, the other 7 primers of every 8 SBE primers were tailed in the 5’ end with three groups of non-homologous polynucleotides of different lengths to facilitate future multiplex genotyping application. All the F, R and tailed SBE primers, 96 each, were synthesized by Eurofins MWG Operon (Huntsville, Al) in a 96-well plate, respectively, where every three primers of each SNP were placed in the same well of the three different plates and stored in ddH_2_O at 10 μM. The format facilitated easy primer positioning and channel pipetting during the genotyping and sequencing preparation.

### SNP 25-nucleotide sequence blast

All 25-nucleotide oligo sequences (SNP in the middle nucleotide) generated from every citrus genotype by QualitySNP were combined together and used to align to the haploid Clementine reference genome (version 1.0; phytozome.org and citrusgenomedb.org) using BLASTN [29] and a cut-off e-value of 6e-004 (0.0006). Each query sequence (25-mer oligo) against the subject scaffolds would yield either of the following BLASTN outputs, “no hits found”, 1 hit on 1 scaffold with 1 alignment, or any other cases (i.e., 1 hit on 1 scaffold with 2+ alignments at different positions or 2+ hits on different scaffolds with 1+ alignment each hit). At the preset e value, only alignments with 84% identities and higher (in other words, only 6 types of alignment hits: 25/25, 24/25, 24/24, 23/23, 22/22, and 21/21), were saved in the BLASTN output file. The information in the output file, including the scaffold, position, strand, e value, score, alignment identities of each hit, and hit status, was parsed into an EXCEL file to summarize SNP alignment status and to calculate distribution on the Clementine reference genome scaffolds. The information was also used as additional criteria for categorization of SNPs and selection of desired core sets.

### SNP validation by sequencing and SNaPshot genotyping assay

BigDye Terminator V3.1 Cycle Sequencing Kit and SNaPshot Multiplex Kit (Applied Biosystems, Foster City, CA) were used to validate SNPs, following the manufacturer’s protocols with some modifications in reaction volumes and/or quantity of proprietary reagents. 96-well plates were used for PCR, enzymatic incubation, and denaturation on iCycler (Bio-Rad, Hercules, CA) and/or GeneAmp PCR System 9700 (Applied Biosystems, Foster City, CA), and for genotyping and sequencing on 3130xl Genetic Analyzer (Applied Biosystems, Foster City, CA). Unless otherwise stated, brief centrifugation up to 1000 rpm in Juan MR 23i was applied after addition of a solution or before implementation of new steps, and all the PCR and enzymatic incubation programs were set to hold at 4°C indefinitely at the end until a next procedure.

For both dye terminator sequencing and SNaPshot assays to validate SNPs, template preparation was carried out in 10 μl in each well consisting of 3.3 μl ddH_2_O, 1.0 μl 10x dNTPs (2 mM), 2.0 μl 5x colorless GoTaq Flexi buffer, 0.8 μl 25 mM MgCl_2_, 0.4 μl F and R primers each, 0.1 μl GoTaq Flexi (5 units per μl Promega, Madison, WI), and 2 μl genomic DNA (10 ng/μl). The touch-down PCR program started from an initial denaturation at 94°C for 3 min, followed by 10 cycles of 93°C for 30 sec, 56°C for 45 sec (decreasing 0.5°C each annealing step), 72°C for 45 sec, and 30 continuing cycles with 51°C at the annealing step, plus a final elongation at 72°C for 15 min. Removal of primers and unused dNTPs was performed by addition of 1 μl of ExoISAP-IT (Affymetrix, Santa Clara, CA) into each well of the plate, and incubation at 37°C for 60 min and 75°C for 15 min.

Sequencing reactions for SNP validation were prepared in 10 μl in each well of a new plate including 2 μl 5x sequencing buffer, 2 μl ready reaction premix in the sequencing kit, 1 μl 10 μM SNP F primer, and 5 μl ExoSAP-IT treated PCR product, started at 95°C for 1 min, followed by 25 thermal cycles of 95°C for 10 sec, 50°C for 5 sec, and 60°C for 4 min. Following the manufacturer’s instructions, ethanol/EDTA/sodium acetate precipitation was used to purify the sequencing product in the plate, which was subsequently air dried, then mixed with 2 μl ddH_2_O and 6 μl Hi-Di formamide in each well, denatured, and loaded to the genetic analyzer to sequence. The sequence files generated were analyzed by Sequencing Analysis software (Applied Biosystems, Foster City, CA) to generate sequences and electropherograms, in which a validated SNP was confirmed by correct alignment of SBE primer sequence into the corresponding sequences and visualization of two different overlapped nucleotide peaks at the nucleotide site in the electropherograms.

The SBE reaction for SNaPshot assays was prepared in 5 μl in each well in a new plate including 0.5 μl ready reaction premix in the SNaPshot kit, 1 μl SBE 10 μM primer, and 3.5 μl ExoSAP-IT treated PCR product, and repeated in 25 thermal cycles of 95°C for 10 sec, 50°C for 5 sec, and 60°C for 30 sec. Removal of unincorporated dye-labeled ddNTPs was completed by addition of 5 μl SAP mix (3.5 μl ddH_2_O, 1.0 μl 10x SAP buffer, and 0.5 μl 1u/μl SAP) into the SBE reaction mix, and incubation at 37°C for 60 min and 75°C for 15 min. Genotyping was performed using 8 μl mix in each well of a new plate consisting of 1 μl SAP treated SBE product, 0.25 μl GeneScan 120 LIZ size standard, and 6.75 μl Hi-Di formamide, which was denatured at 95°C 3 min then immediately moved on ice for at least 2 min. The SNaPshot files were used to score SNPs by GeneMarker (SoftGenetics, State College, PA) in which a validated SNP consisted of two different nucleotides.

## Results

### Haplotype-based EST-SNPs in citrus cultivars

**Figure 1:**
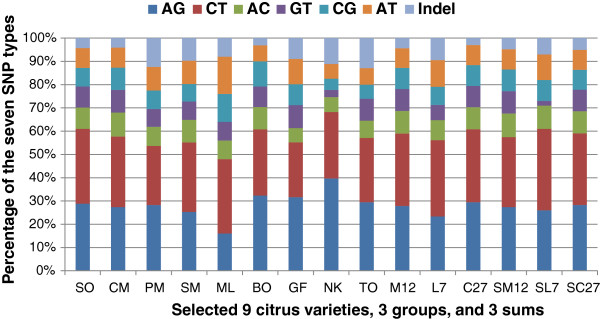
**Percentages of the 7 SNP types, AG, CT, AC, GT, CG, AT, and indel, discovered from citrus ESTs.** Presented here are 9 selected citrus cultivars, 3 groups, and 3 sums. SO, Sweet orange; CM, Clementine mandarin; PM, Ponkan mandarin; SM, Satsuma mandarin; ML, Rangpur lime; BO, Sour orange; GF, Grapefruit; NK, Nagami kumquat; TO, Trifoliate orange; M12, SNPs from ESTs combined from 12 mandarins (2–13 in Table 1), L7, SNPs from ESTs combined from 7 limes / lemons (14–20 in Table 1); C27, SNPs from all ESTs combined (1–27 in Table 1); SM12, SL7 and SC27, the respective sum of the 12 mandarins, 7 limes/lemons, and all 27 cultivars. On the average of the 27 cultivars (SC27), transitions (AG and CT) account for 59.1%, transversions (AC, GT, CG, and AT) for 35.9%, and insertion/deletions (indels) for 5.0%.

Haplotype-based SNPs were mined from ESTs of the 27 citrus cultivars and 3 groups (M12 – 12 mandarins, L7 – 7 limes/lemons, and C27 – all 27 combined) using the QualitySNP pipeline and summarized in detail (Additional file 1). In summary (SC27 – the last column in Additional file 1), a total of 25,417 qSNPs (Additional file 2) were identified from ESTs of the 27 cultivars mined separately. These are attributed to heterozygosity within cultivars at SNP loci. There were only 2805 SNPs duplicated according to comparison of all the 25-mer oligo sequences. The percentages of the 7 SNP types were similar among most citrus cultivars with each type of quality SNPs found. Among the 25,417 qSNPs summed from the 27 citrus cultivars, 15,010 (59.1%) were transitions (AG and CT), 9,114 (35.9%) transversions (AC, GT, CG, and AT), and 1,293 (5.0%) insertion/deletion events (indels). On average, there were 2.4 SNPs per contig and one SNP every 1,064 bp in all of the SNP-containing contig sequences (Figure 1; Additional file 1).

For individual cultivars, their numbers of ESTs were different, so consequentially were their quality SNPs and other related numbers. For example, in SO, 213,830 ESTs yielded 7,404 contigs of >=4 ESTs. Of these, 4,228 contigs contained 43,655 potential SNPs and 3,327 contained qSNPs. The total number of qSNPs was 11,182. In other words, there was only one haplotype detected in 3,176 contigs (7,404 minus 4,228) and no quality SNP identified in the additional 1,001 contigs (4,428 minus 3,327) with potential SNPs. There were 3.4 quality SNPs per contig and one quality SNP per 723 bp in the contigs on average. Of these 11,182 qSNPs, 6,822 (61.0%) were transitions (AG and CT type), 3,879 (34.7%) transversions (AC, GT, CG, and AT type), and 481 (4.3%) insertion/deletion (Indels); and 2,619 (23.4%) were nsSNPs and 4,038 (36.1%) were sSNPs. The absolute numbers of quality SNPs were not comparable due to varying numbers of ESTs among citrus cultivars, but the number of potential and quality SNPs from each cultivar were strongly correlated with its number of ESTs; more ESTs yielded more usable contigs (>=4 ESTs) available for SNP mining, as well as more quality SNPs (Additional file 1). Given the large differences in the numbers of ESTs available among the various cultivars, it is more interesting to compare SNP frequencies, rates, and ratios among cultivars with substantial EST numbers and distinct genetic backgrounds, and differences between the mining results of the three grouped ESTs (M12, L7, and C27) and the three sums/averages (SM12, SL7, and SC27) of separately mined counterpart individuals. These comparisons will be elaborated hereafter.

### Haplotypes detected in contigs with SNPs

**Figure 2:**
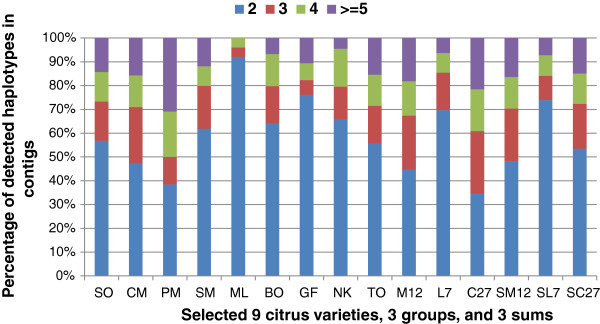
**Percentages of detected haplotype numbers (2, 3, 4, and >=5) in contigs (>=4 ESTs) with potential SNPs.** Presented here are 9 selected citrus cultivars, 3 groups, and 3 sums. SO, Sweet orange; CM, Clementine mandarin; PM, Ponkan mandarin; SM, Satsuma mandarin; ML, Rangpur lime; BO, Sour orange; GF, Grapefruit; NK, Nagami kumquat; TO, Trifoliate orange; M12, SNPs from ESTs combined from 12 mandarins (2–13 in Table 1), L7, SNPs from ESTs combined from 7 limes/lemons (14–20 in Table 1); C27, SNPs from all ESTs combined (1–27 in Table 1); SM12, SL7 and SC27, the respective sum of the 12 mandarins, 7 limes/lemons, and all 27 cultivars.

**Figure 3:**
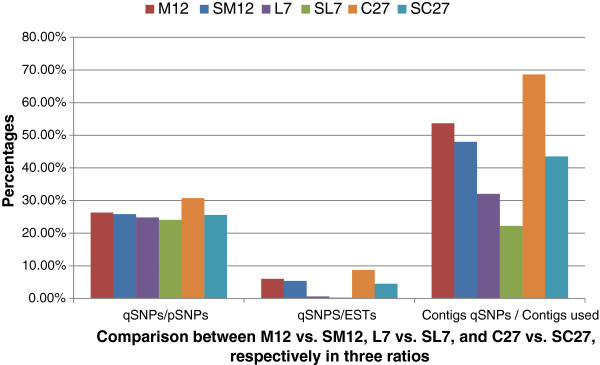
**Comparisons between M12 vs. SM12, L7 vs. SL7, and C27 vs. SC27, respectively in three ratios.** There are three ratios presented as percentage, qSNPs, the number of quality SNPs; pSNPs, the number of potential SNPs; ESTs, the number of ESTs; contigs qSNPs, the number of contigs with qSNPs; contigs used, the number of contigs with >=4 ESTs. M12, L7 and C27 are mined from grouped ESTs from the corresponding cultivars, and SM12, SL7, and SC27 summed from individually mined cultivars used in the grouped counterparts, respectively.

One important feature of QualitySNP is to re-cluster ESTs in a contig to reconstruct and determine the haplotypes in that contig, from which only single nucleotide discrepancies between any two defined haplotypes (allelic sequences) are considered as potential SNPs for further quality and confidence interrogation. Only those potential SNPs passing confidence scores are identified as quality SNPs. In Additional file 1, all the haplotypes detected in the SNP-containing contigs from all the 27 citrus cultivars are included. Theoretically, there should be only a maximum of 2 haplotypes detected in a diploid genome. As expected, a vast majority of SNP-containing contigs consisted of two haplotypes, but the percentages of 2 haplotypes varied in a wide range in these citrus cultivars (Figure 2, Additional file 1). Among the highest were ML (92%), SC (84%), and GF (76%), and among the lowest PM (38%), KL (42%), and CM (48%). The variation likely results from the genetic makeup of the “cultivar” used to generate the ESTs. For example, ESTs for SO came from navel oranges, blood oranges, and others named *C. sinensis*, rather than a single genotype. In contrast, other “cultivars” are likely single clones. It was also evident as expected that much lower percentages of 2 haplotypes were found in three combined EST datasets (M12, 44%; L7, 70%; and C27, 34%) due to introduction of more haplotypes from different types of citrus cultivars, compared to their counterpart averages of each group (SM12, 48%; SL7, 74%; and SC27, 53%). As a consequence, more qSNPs in higher qSNPs/pSNPs and qSNPs/ESTs ratios were found in the three grouped EST datasets (M12, L7, and C27), compared to their counterparts (SM12, SL7, and SC27) summed from the individually mined cultivar EST results, but the ratio of contigs with qSNPs and contigs used was the opposite (Figure 3, Additional file 1). The frequency of qSNPs is much higher in the pooled data for the three groups (M12, L7 and C27) than in the summed data for individual cultivars. This is because the group values include polymorphism among homozygous accessions as well as heterozygosity within cultivars, while the summed data include only SNPs due to heterozygosity. In other words, the nucleotide at such a SNP is very likely homozygous within a genotype, making it useless in genetic linkage mapping of that genotype.

### Alignment and distribution on the Clementine reference genome

A total of 25,417 25-mer sequences (query sequence, Additional file 2) with quality SNPs from all the 27 citrus cultivars were used to align to the Clementine reference scaffolds (subject sequence) using BLASTN at a cut-off e-value of 6e-004 (Table 2). 2,947 sequences had “no hits found” and 22,470 one or more hits. Of the 22,470 SNPs with hits, 19,943 had only 1 scaffold hit with only 1 alignment on the scaffold, 1,571 had 1 scaffold hit but >=2 alignments on the scaffold (3 alignments per scaffold hit on average), and 956 had >=2 scaffold hits (~3 hits per oligo on average) with 1 or more alignments on each of the scaffolds (~7 alignments per scaffold hit or ~20 alignments per oligo on average). It suggested the 19,943 25-mer oligo sequences appear to be unique in the genome, and the remaining 2,527 25-mer sequences may have duplicated or similar sequences with at least 84% identities at different locations in the genome. There was one extreme case that one 25-mer sequence from trifoliate orange yielded 29 scaffold hits and 2,162 alignments on all the scaffolds, the highest numbers of all.

**Table 2:** BLASTN results of 25,417 25-mer oligo sequences

	**25-mers**	**Hits**	**Alns**	**25/25**	**24/25**	**24/24**	**23/23**	**22/22**	**21/21**
No hits found	2,947								
1 hit (1 aln)	19,943	19,943	19,943	10,926	8,555	127	116	112	107
1 hit (2+ aln)	1,571	1,571	4,614	2,152	2,026	161	73	78	124
2+ hits (1+ aln each hit)	956	2,779	19,111	7,923	9,014	389	353	715	717
Total	25,417	24,293	43,668	21,001	19,595	677	542	905	948

The Clementine reference genome was used as the BLAST database. All the oligo sequences were listed in Additional file 2. Aln – Alignment(s); 1 hit (1 aln) – hit only 1 scaffold with 1 alignment; 1 hit (2+ aln) – hit on only 1 scaffold but with 2 or more alignments, and 2+ hits (1 + aln) – hit on 2 and more scaffolds with one or more alignment to each scaffold.

**Figure 4:**
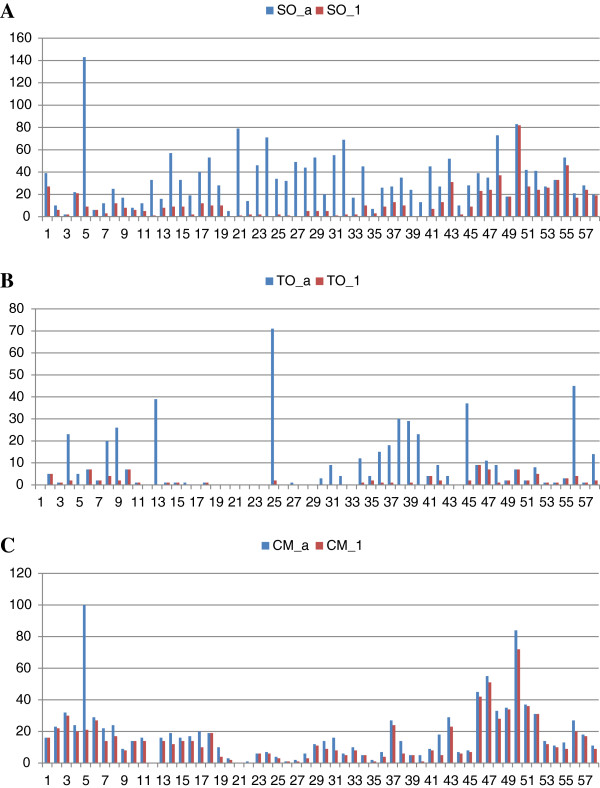
**SNP distribution on the Clementine reference genome, using Scaffold_1 as an example.** Each interval of the x-axis represented 500 kb of the scaffold, and the y-axis represented the number of SNPs in each 500 kb on the scaffold. SO – sweet orange **(A)**; TO – trifoliate orange **(B)**; CM – Clementine mandarin **(C)**; “_a” – counts of all alignments generated by all SNPs; “_1” – counts of SNPs of only 1 unique hit/alignment in the genome. Differences between the “_a” and “_1” numbers are observed in several regions of each cultivar.

Taking these multiple scaffold hits and alignments into account, the total number of scaffold hits was 24,293 with a total of 43,668 alignments on the scaffolds. Most had 100% (25/25) or 96% (24/25) nucleotide identities to those on the reference genome, accounting for 93% of all the alignments. Almost all the nucleotide discrepancies in the 24/25 alignments were at the SNP sites, which is an encouraging *in silico* validation of these SNPs. Of the total 24,293 scaffold hits, 23,955 were on main scaffolds 1 to 9 (2,122, 2,804, 4,159, 2,813, 3,045, 2,501, 1,861, 2,308, and 2,342, respectively), accounting for 98.6% of the total. The remaining 338 were on 87 small scaffolds. Figure 4 showed the distribution of SNPs with all and unique hits from SO, TO, and CM on scaffold_1 of the haploid Clementine genome (similar figures on scaffold_2 are in Additional file 3). According to the aligned SNP counts on each 500 kb, there were some featured regions (intervals in Figure 4). For example, in SO many fewer unique hits were found in the middle region, compared to those in two arm regions. Relatively even distribution was observed in CM, with exceptions at Interval 5 with overwhelming duplicated hits of certain SNPs (similar to the same region in SO). There were very limited unique SNPs aligned at Interval 20–27 of all the three cultivars, suggesting the region may contain the centromere, usually characterized by fewer genes. These results, combined with other criteria, should greatly facilitate selection of well-distributed core sets of SNPs across citrus genomes for different genotyping applications and genetic studies.

### SNP validation by sequencing and SNaPshot genotyping assay

Of the 96 randomly selected sweet orange SNPs, 68 were validated by sequencing and 74 by SNaPshot in sweet orange (Additional file 4). There were 61 validated by both assays and the remainder validated by only one assay. In other words, 7 were validated by only sequencing but failed in SNaPshot, and 13 by only SNaPshot but failed in sequencing. Therefore, a total of 81 SNPs (84%) were validated by at least one of the two assays. The high rate (84%) of validated SNPs was consistent with 93% alignments onto the reference genome with 100% (25/25) or 96% (24/25) identities (Table 2), indicating that QualitySNP, a haplotype-based SNP mining algorithm and pipeline, is a very reliable tool to identify true EST SNPs, and it can effectively minimize the false discovery rate even without quality files.

## Discussion

### Estimation of heterozygosity of different citrus genomes by haplotype-based SNPs

Many naturally evolved genomes are heterozygous, and the heterozygosity level may be evaluated by the rate of allelic nucleotide variations between the two haplotypes [30]. SNPs, the most abundant polymorphisms in genomes, likely are the most appropriate index for the heterozygosity levels of genetically/taxonomically related genomes [19,21,22]. Given the different numbers and rates of haplotype-based SNPs discovered from these citrus individuals with substantial numbers of ESTs (for example more than 5,000, Additional file 1), the ratios of qSNPs/ESTs in most of them appeared reflective of their heterozygous status and genetic background. These hybrid derivatives had much higher qSNPs/ESTs ratio, while the other believed “pure” species had lower ratios. For example, some proven natural hybrid cultivars, such as SO, CM, and recent hybrids such as SC, were among the higher qSNPs/ESTs ratios (SO - 5.23%, CM - 8.31%, and SC - 7.76%). Other presumed true species, including PM, fell in the lower qSNP/ESTs ratios (PM - 0.60%). The number of needed ESTs to generate the desired number of SNPs in given citrus genotypes, and vice versa, can be estimated. Such a tendency, along with the ratios and genome heterozygosity, could be strengthened and would be more conclusive if the numbers of ESTs in all the cultivars were close to each other, or at least in a much smaller range.

### SNP discovery and validation rates

SNP mining is no longer a bottleneck because computational capacity and sequence data are exponentially increasing, and more SNP mining pipelines have become available in recent years [7,8,12-15,31]. Hundreds of thousands of SNPs can be easily mined out of EST or genomic sequences. Inclusion of false SNPs in genotyping certainly is wasteful; therefore, maximizing the true SNP rate (minimizing the false rate) is the most important consideration or requirement for a SNP mining algorithm because any validation approach can only validate these true SNPs, but not false ones [8,13]. We found that 93% of SNPs identified by the QualitySNP pipeline were aligned onto the reference genome at 25/25 or 24/25 identities, and 81% of randomly selected sweet orange SNPs were validated by sequencing and SNaPshot genotyping. It was undetermined whether the others not aligned at the two identity rates, and not validated by sequencing and/or genotyping, were true or false SNPs. For example, those failing in sequencing validation might be due to SBE primer sequences not being found (likely an intron in the region), or sequencing failures caused by primers of low quality or in a variable region, or no nucleotide discrepancies at the sites. It was unclear how these SNPs failed in SNaPshot validation; it is speculated some of these SBE primers might be incorrectly positioned, i.e., the singly extended nucleotides may not have been exactly at the SNP sites. There were a few such cases identified (Chen et al. unpublished data); very likely due to the differences between these consensus contigs and the original haplotype sequences. On the other hand, only 2 haplotypes may exist in a diploid genome. If SNPs were from the contigs with more than 2 haplotypes, such cases could result from either ESTs mixed from diverse genotypes in the same species or highly identical paralogs assembled into the contigs. Paralogous genes, resulting from genomic duplication and evolving into different functions, are very common in many genomes and remain almost identical in their conserved regions. ESTs from different paralogous genes, if assembled into a same unigene, could yield false SNPs that are non-allelic and useless.

### Criteria for selection of citrus core SNP sets

In most cases the discovered SNPs could easily reach a number so large that only a small portion of them, designated core SNP set, are selected and used in genotyping to meet the restraints in available budget, desired platform, applications, and other factors [3,11,32-34]. These core sets of different numbers (e.g. 384, 1536, or other numbers) are either required by certain SNP genotyping platforms or optimized for particular applications [35-38]. It may be a daunting job, but it is necessary to establish workable criteria to select any core set of different numbers of SNPs. Based on this complete mining and validation process, several attributes of SNPs can be very useful and distinguishing to refine these core sets of different numbers. SNP oligo alignment uniqueness, identity percentage, and distribution in the reference genome, co-existence across different genomes, along with SNP types (nsSNP vs. sSNP, and transition vs. transversion vs. indel) and numbers per gene, should be the main criteria for selection of citrus core SNP sets. As pointed out, some extra haplotypes might result from paralogs across different genome regions. In that case, the resulting SNPs would not be allelic or useful. Whether they mostly were those SNPs that had multiple scaffold hits and alignments remains unclear pending further investigation. Those SNPs from either circumstance should be excluded or at least deprioritized for use in genotyping. Selection of SNPs for genotyping could be difficult when different attributes of SNPs and genotyping platforms are considered. A tool based on these attributes is being developed to achieve the automatic selection of core SNP sets for targeted applications/platforms [35,36] and to allow geneticists and molecular breeders to be able to select and use certain core SNPs of interest from among the thousands discovered [37,38]. All the SNPs (Additional file 2) identified in this work are being added to a citrus genome database (citrusgenomedb.org). Very recently after this study, another draft genome of sweet orange was reported, yielding 1.06 million genome-wide SNPs, about 3.6 SNPs/kb, which could be an additional valuable resource in SNP applications [39].

## Conclusions

High-quality SNPs in public ESTs from different citrus genotypes were detected by the QualitySNP pipeline and compared to estimate the heterozygosity of each genome. All the short SNP oligo sequences were also aligned with the Clementine citrus genome to determine their distribution and uniqueness in the genome and for in silico validation. Selected SNPs were also validated by SNaPshot and sequencing.

## Competing interests

The authors declare that they have no competing interests.

## Authors’ contributions

CC conceived the study, performed bioinformatics analysis and comparison, and wrote the manuscript, and FG critically read and revised the manuscript. All the authors read and approved the final manuscript.

## Supplementary Material

Additional file 1: Table S1 Summary of citrus EST SNPs. It includes mining results from 27 individual varieties with their index number, binomial name, common name, and abbreviation, 3 grouped ESTs - M12, 12 mandarins (2–13); L7, 7 limes/lemons (14–20); C27, all 27 citrus varieties (1–27); and three summed/averaged results, SM12, SL7 and SC27, respectively from the 12 individually mined mandarins, 7 limes/lemons, and all 27 varieties, which were used for comparison to M12, L7, and C27. Click here for file

Additional file 2: Table S2 25417 25-mer sequences of SNPs and forward, reverse, single base extension (SBE) primer, and SBE 5'‒tail sequences for 96 SNPs selected from sweet orange. Click here for file

Additional file 3: Figure S1 SNP distribution on the Clementine reference genome Scaffold_2. Each interval of the x-axis represented 500 kb of the scaffold, and the y-axis represented the number of SNPs in each 500 kb on the scaffold. SO – sweet orange (A); TO – trifoliate orange (B); CM – Clementine mandarin (C); “_a” – counts of all alignments generated by all SNPs; “_1” – counts of SNPs of only 1 unique hit/alignment in the genome. Differences between the “_a” and “_1” numbers were observed in several regions of each cultivar. Click here for file

Additional file 4: Figure S2 SNapShot chromatograph of a SNP validated by the assay, generated by GeneMarker (SoftGenetics, State College, PA). The y-axis represents the intensity of, and x-axis the approximate length of, the fluorescently-labeled SBE products ending with A and G. Click here for file
