# Supplementary material for: Mining of haplotype-based expressed sequence tag single nucleotide polymorphisms in citrus
Source: BMC Genomics. 2013 Nov 1;14:746. doi: 10.1186/1471-2164-14-746 (PMC3827885; doi:10.1186/1471-2164-14-746)
Supplement: Additional file 4: Figure S2 — SNapShot chromatograph of a SNP validated by the assay, generated by GeneMarker (SoftGenetics, State College, PA). The y-axis represents the intensity of, and x-axis the approximate length of, the fluorescently-labeled SBE products ending with A and G. [file 1471-2164-14-746-S4.pdf]

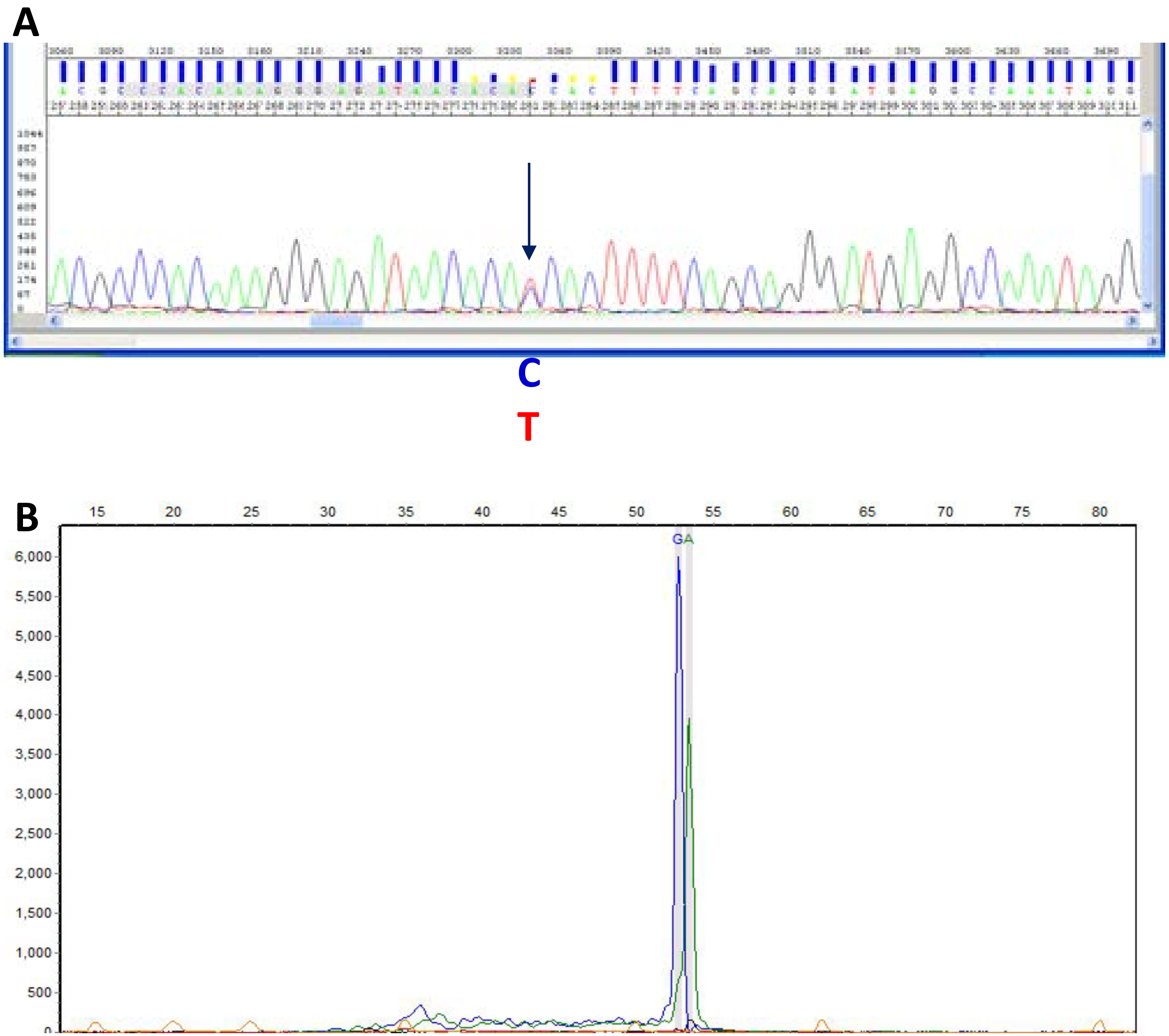

Figure S2. Example of SNP validation by sequencing (**A**) and SNaPshot (**B**). In **A**, a proprietary sequencing file was generated by a ABI sequencer and analyzed by Sequencing Analysis software (Applied Biosystems, Foster City, CA) to generate the sequence and electrophoregram, in which the validated SNP was confirmed by visualization of overlapping of the two nucleotides (C/T) at the SNP site and alignment of its SBE primer sequence. In **B**, a SNaPshot chromatograph of a SNP validated by the assay was generated by GeneMarker (SoftGenetics, State College, PA), revealing the two nucleotides (A/G) at the SNP site. The y-axis represents the intensity of, and x-axis the approximate length of, the fluorescently-labeled SBE products ending with A and G.
