# Supplementary material for: Mining of haplotype-based expressed sequence tag single nucleotide polymorphisms in citrus
Source: BMC Genomics. 2013 Nov 1;14:746. doi: 10.1186/1471-2164-14-746 (PMC3827885; doi:10.1186/1471-2164-14-746)
Supplement: Additional file 3: Figure S1 — SNP distribution on the Clementine reference genome Scaffold_2. Each interval of the x-axis represented 500 kb of the scaffold, and the y-axis represented the number of SNPs in each 500 kb on the scaffold. SO – sweet orange (A); TO – trifoliate orange (B); CM – Clementine mandarin (C); “_a” – counts of all alignments generated by all SNPs; “_1” – counts of SNPs of only 1 unique hit/alignment in the genome. Differences between the “_a” and “_1” numbers were observed in several regions of each cultivar. [file 1471-2164-14-746-S3.pdf]

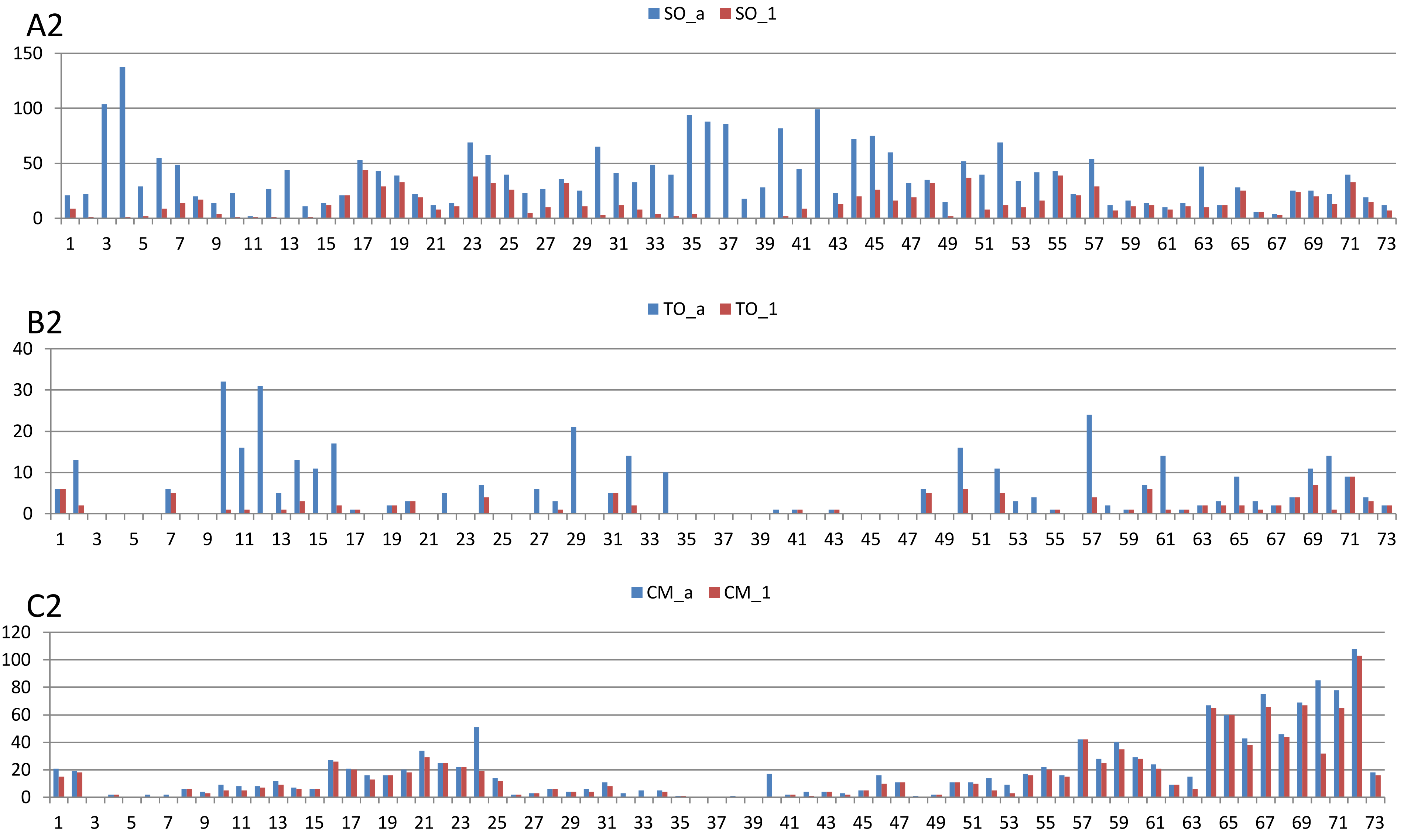

Additional File 3 Figure S1. SNP distribution on the Clementine reference genome Scaffold_2. Each interval of the x-axis represented 500kb of the scaffold, and the y-axis represented the number of SNPs in each 500kb on the scaffold. SO – sweet orange (**A2**); TO – trifoliate orange (**B2**); CM – Clementine mandarin (**C2**); "_a" – counts of all alignment generated by all SNPs; "_1" – counts of SNPs of only 1 unique hit/alignment in the genome. Differences between the "_a" and "_1" numbers were observed in several regions of each cultivar.

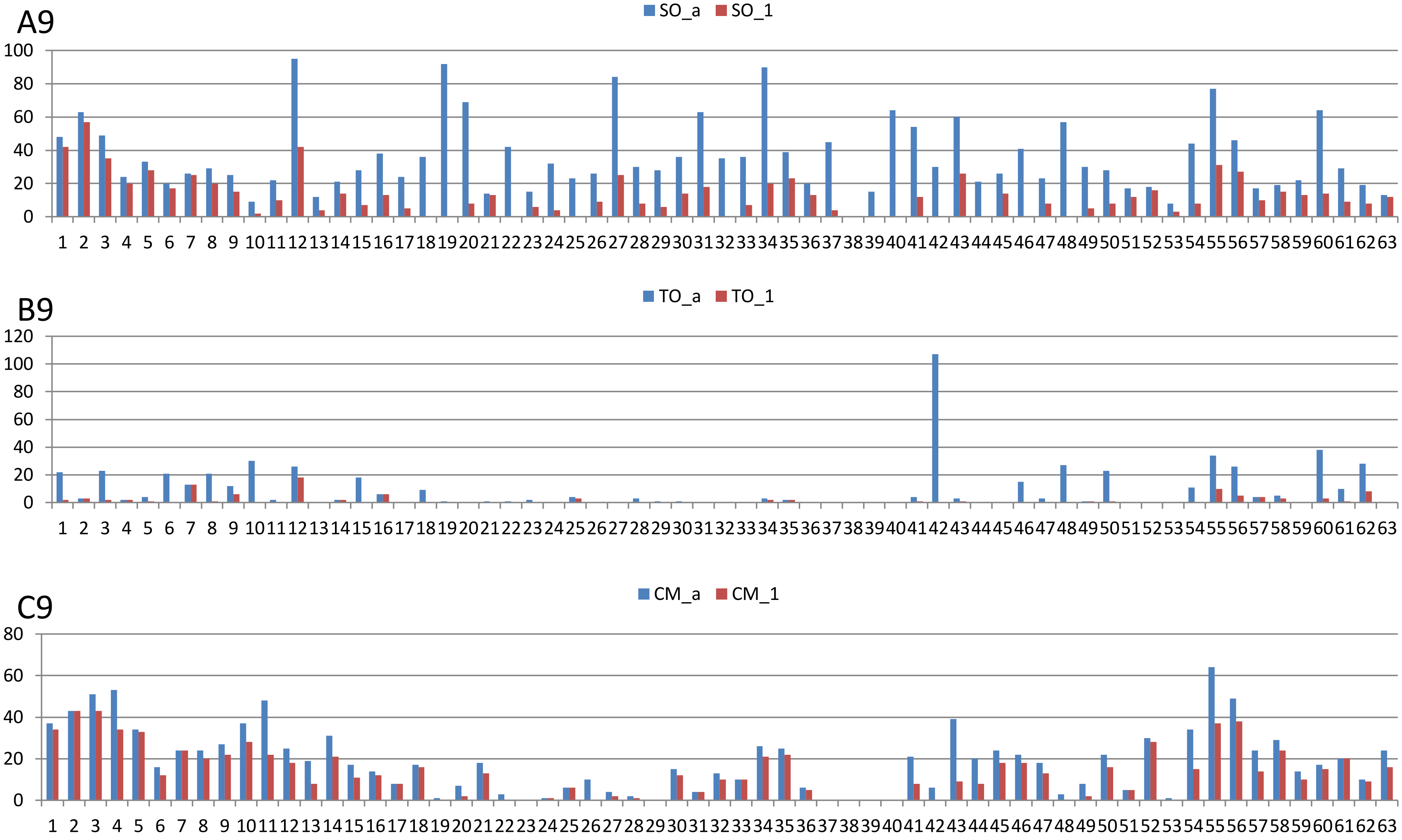

Additional File 3 Figure S1. SNP distribution on the Clementine reference genome Scaffold_9. Each interval of the x-axis represented 500kb of the scaffold, and the y-axis represented the number of SNPs in each 500kb on the scaffold. SO – sweet orange (**A9**); TO – trifoliate orange (**B9**); CM – Clementine mandarin (**C9**); "_a" – counts of all alignments generated by all SNPs; "_1" – counts of SNPs of only 1 unique hit/alignment in the genome. Differences between the "_a" and "_1" numbers were observed in several regions of each cultivar.
